# Inhibitory Effects of Pulse Bioactive Compounds on Cancer Development Pathways

**DOI:** 10.3390/diseases6030072

**Published:** 2018-08-03

**Authors:** Shiwangni Rao, Kenneth A. Chinkwo, Abishek B. Santhakumar, Christopher L. Blanchard

**Affiliations:** 1 School of Biomedical Sciences, Charles Sturt University, Wagga Wagga, NSW 2650, Australia; srao@csu.edu.au (S.R.); kchinkwo@csu.edu.au (K.A.C.); CBlanchard@csu.edu.au (C.L.B.); 2 Australian Research Council (ARC) Industrial Transformation Training Centre (ITTC) for Functional Grains, Graham Centre for Agricultural Innovation, Charles Sturt University, Wagga Wagga, NSW 2650, Australia

**Keywords:** pulses, anti-cancer, antioxidant, polyphenols, peptides

## Abstract

Previous studies suggest that pulses may have the potential to protect against cancer development by inhibiting pathways that result in the development of cancer. These pathways include those that result in inflammation, DNA damage, cell proliferation, and metastasis. Other studies have demonstrated extracts from pulses have the capacity to induce apoptosis specifically in cancer cells. Compounds reported to be responsible for these activities have included phenolic compounds, proteins and short chain fatty acids. The majority of the studies have been undertaken using in vitro cell culture models, however, there are a small number of in vivo studies that support the hypothesis that pulse consumption may inhibit cancer development. This review highlights the potential benefit of a diet rich in pulse bioactive compounds by exploring the anti-cancer properties of its polyphenols, proteins and short chain fatty acids.

## 1. Introduction

Pulses are edible seeds that belong to the legume family Fabaceae/Leguminosae, and are used for human consumption or as animal feed. The Food and Agriculture Organisation (FAO) listed 11 important types of pulses consumed around the world, including dry beans, dry broad beans, dry peas, chickpeas, dry cowpea, pigeon pea, lentil, groundnut, vetches, and lupin [1]. They are consumed for their high protein levels and carbohydrate rich endosperm. Pulses have also been demonstrated to contain bioactive compounds that alleviate risk factors associated with diabetes [2], metabolic syndrome [3], inflammation [4], and cancer [5,6].

There has been considerable interest in exploring the chemo-preventive properties of pulses and/or their derivatives using both in vitro and in vivo cancer models [7]. Some of the potential health beneficial properties of pulses have been attributed to the antioxidant activity exhibited by phenolic compounds [8,9], and/or the mechanistic involvement of proteins [10] and short chain fatty acids [11] in the disease process. This paper aims to review current literature describing the potential of pulses as effective anti-cancer nutraceutical agents. Specifically, the role that pulse derived phenolic compounds, proteins and fatty acids play in mechanistic pathways of anti-inflammatory, anti-apoptotic, anti-proliferative and anti-metastatic will be discussed.

## 2. Bioactive Compounds in Pulses

**Figure 1:**
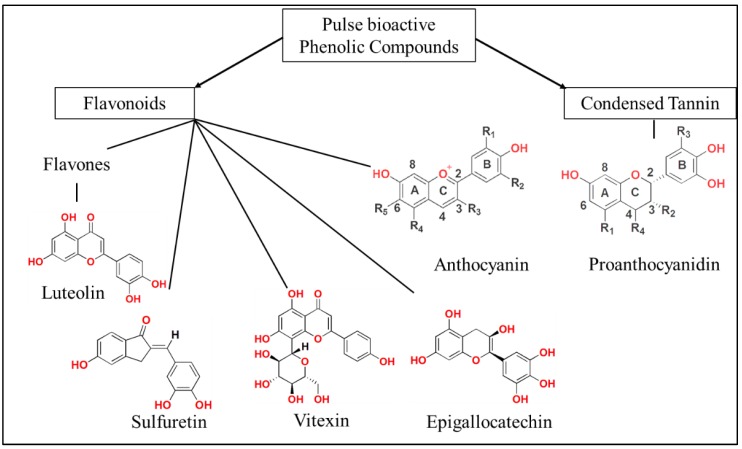
Bioactive flavonoids, anthocyanins, and tannins found in pulses.

Pulses have a seed anatomy consisting of the endosperm, seed coat, and embryo (ridicule and root). The endosperm, which is the primary biomass of the seed, contains carbohydrates, storage proteins and fat. The seed coat of pulses are often rich in phenolic compounds [12], and studies have identified the presence of flavonoids, anthocyanins and tannins [12,13] (Figure 1). These phenolic compounds have high antioxidant activity [13] and their ability to scavenge free radicals has been correlated to anti-inflammatory [14,15], anti-proliferative [9] and anti-apoptotic activity. While crude extracts from pulses have been demonstrated to exhibit antioxidant activity, some pulse derived phenolic compounds such as anthocyanins [16] and iso-flavanols [17] have been demonstrated to be effective in targeting specific cancer pathways.

Pulses contain proteins which are often classified as albumins, globulins, glutelins, and prolamins [18]. Some specific types of proteins such as lectins (carbohydrate-binding proteins) have been shown to exhibit anti-cancer properties [19]. For example. lectins derived from pulses are thought to bind to tumour membranes, blunt cellular proliferation, stimulate the immune system and induce apoptosis. Furthermore, protein protease inhibitors such as Bowman-Birk inhibitors (BBI), have been reported to target specific anti-cancer pathways [20]. Protease inhibitors have been found to have potential anti-cancer properties in in vitro and in vivo experiments by inhibiting the generation of reactive oxygen species. Pulses generally have low levels of lipids, however, fatty acids such as butyrate, oleic and linoleic acid have been associated with anti-cancer activities [11,21,22].

## 3. Cancer Development and Mechanisms of Inhibition

Transformation of normal cells into malignant cells due to mutations, result in genome instability of DNA molecules [23]. The mutated cancer cells develop salient characteristics to evade the immune system, sustain proliferative signalling, activates invasion with metastasis, resist cell death and initiate tumour promoting inflammation [23]. It has been reported that DNA damage from free radicals, increase cell cycle replication error from faulty tumour suppressors and inactivated repair machinery [24]. This scenario provides the right conditions to alter the mechanisms that regulate proliferation and apoptosis in normal cells with subsequent transformation into cancer cells.

By targeting specific points in cancer development pathways, pulse bioactive compounds have the potential to reduce the initiation and progression of cancer development and possibly play a role in the elimination of malignant cells. Incorporating pulses in diets have been shown to have beneficial effects against chronic conditions [25]. Correlations have been identified between pulse consumption and a reduction in inflammation and cancer development [26]. Studies have identified the anticancer potential of pulses including adzuki bean, black bean, mung bean, pinto bean, faba beans, lupins, lentils and chickpeas (Table 1 and Table 2). It has been proposed that these pulses contain phenolic compounds, proteins and short chain fatty acids with the capacity to deter the progression of cancer cells development. These pulse bioactive compounds are thought to inhibit specific pathways to reduce cancer development by reducing inflammation, cancer cell proliferation and induce apoptosis specifically in cancer cells.

### 3.1. Anti-Inflammation

#### 3.1.1. Role of Pulse Phenolic Compounds

Inflammation is the body’s natural response mechanisms against foreign antigens. However, cancer-related inflammation has a cascading effect that leads to tumourigenesis [27]. Oxidative stress due to the presence of free radicals activates the enzyme IkB Kinase (IKK) which initiates the cellular nuclear factor kappa β (NF-κβ) pathway [28]. This results in other signal transduction pathways being activated such as the mitogen-activated protein kinase (MAPK) pathway which has been reported to promote cancer-related inflammation [27]. The activation of these pathways results in the production of cytokine signaling molecules/mediators such as cyclooxygenase (COX), tumour necrosis factor alpha (TNF-α), and nitric oxide (NO). This, in turn, initiates the production of interleukin (IL), prostaglandin E_2_ (PGE_2_) and site-specific macrophage adhesion [29,30]. This disruption of the signaling network promotes the neighboring cells and tissue to support tumourigenesis [28].

Several studies have shown that phenolic compounds extracted from pulses have anti-inflammatory properties and free radical scavenging activity [31,32]. An in vitro experiment was conducted to investigate the anti-inflammatory effect of four bean varieties using colorectal cancer cell lines [32]. It was observed that all the bean extracts exerted an anti-inflammatory effect. It was confirmed that the level of the anti-inflammatory response was correlated to the anti-proliferative effect. Bean extracts were observed to inhibit pro-inflammatory proteins such as cyclooxygenase-2 (COX-2), tumour necrosis factor α (TNFα), and nuclear factor kappa β (NF-κβ) while increasing the activity of interleukin 10 (IL-10), an anti-inflammatory protein. In another experiment, Faba beans inhibited inflammation via pro-inflammatory signal transduction pathway, mediated by Lipoxygenase (LOX) [14]. Furthermore, investigations were conducted on anti-inflammatory properties of phenolic extracts from mung bean cotyledon, hull, and whole grain [31]. It was observed that the hull with higher phenolic content and antioxidant activity had the highest anti-inflammatory effect. Lipopolysaccharide (LPS) stimulated RAW 264.7 mouse macrophage cells have also been reported to possess high anti-inflammatory properties in phenolic extracts from wholegrain mung bean [8].

#### 3.1.2. Role of Pulse Bioactive Peptides and Amino Acids

Pulse proteins, peptides and amino acids have been demonstrated to exhibit anti-inflammatory effects. Protein hydrolysates from germinated beans have exhibited an anti-inflammatory effect in RAW 264.7 mouse macrophage cells by reducing nitric oxide synthesis [33]. Also, a non-protein amino acid, gamma-aminobutyric acid (GABA) has been shown to demonstrate anti-inflammatory activity. In vivo experiments using GABA-rich extracts were conducted on female Balb/c mice inoculated with Yac-1 and 4T1 carcinogenic cell lines [34]. Mice supplemented with diets of either 200 mg/kg or 1000 mg/kg body weight of GABA exhibited delayed tumour formation. This was correlated with high anti-cancer cytokine levels, splenic T cell populations, splenocyte cytotoxicity and spleen/tumour antioxidant levels. Mitotic divisions in spleen were also reduced which was accompanied by a reduction in antioxidant and nitric oxide levels [34].

### 3.2. Anti-Proliferation

#### 3.2.1. Role of Pulse Phenolic Compounds

Several studies have investigated the anti-cancer potential of pules by demonstrating its anti-proliferative capacity in cancer cells [6,14,16]. Inhibition of cancer cell line proliferation was examined using phenolic extracts from pulses including green pea, yellow pea, chickpea, lentil, yellow soybean, black soybean, pinto bean, black bean, small red bean, red kidney bean, mung bean, adzuki bean and black-eyed peas on nine cancer cell lines [38]. The study found considerable variability in phenolic profiles and the effect these have on the various cell lines. Adzuki exhibited the highest anti-proliferative effect in a dose-dependent manner in cell lines CAL27, AGS, HepG2, SW480, Caco-2, DU 145, SK-OV-3, MCF-7 and HL-60. Inhibition of proliferation was observed at IC_50_ 0.32, 0.68, 0.36, 0.4, 0.41, 1.98, 0.79, 0.86 and 0.55 mg/mL respectively. Meanwhile, the black-eyed pea, green pea, yellow pea and chickpea extracts did not establish an IC_50_ value, with substantial anti-proliferative effect in the cell lines HepG2, Caco-2, DU145 and SK-OV-3 [38].

Another study examined the effect of phenolic extracts from raw, cooked and fermented Canavalia beans of two types, on HT29 and MCF7 cell lines [39]. It was found that the fermented *Canavalia cathartica* and cooked *Canavalia maritima* were more effective in inhibiting cell proliferation compared to their raw forms. The study deduced that processing by cooking and fermentation significantly increased anti-proliferative properties in canavalia beans. Meanwhile, phenolic compounds extracted from sprouted and dry mung beans have also demonstrated anti-proliferative effects [40]. The study extracted phenolic compounds with methanol, ethanol, hexane, water and butanol and tested their effect on Calu6 and SNU601 cell lines. It was observed that ethanol extracts and extracts from sprouts were more effective at inhibiting proliferation with extracts from sprouted beans having higher anti-proliferative effects.

The effects of pulse pigmentation in relation to inhibition of cancer cell lines proliferation has also been examined [41]. The study used phenolic extracts from 12 pigmented and non-pigmented varieties of bean which were tested using Caco-2, MCF7 and A594 cancer cell lines. It was observed that pigmented varieties were more toxic to cancer cells compared to non-pigmented varieties, with Cannellino Ross having the highest cytotoxicity. While the aforementioned studies have investigated effects of crude phenolic extracts, some studies have identified specific phenolic compounds with anti-proliferative effects [5,42]. These studies identified condensed tannins, isoliquiritigenin, 6,4′-dihydroxy-3′-methoxyaurone, and sulfuretin as possible cytotoxic phenolic compounds present in red bean and tokan bean. In addition, an in vivo study demonstrated female Sprague Dawley rats to have reduced incidences of mammary cancer, multiplicity and tumour size, when fed with bean extracts [35]. Rat diets consisted of 60% bean extract from white kidney, dark red kidney, great northern, small red, navy and black bean. In contrast, to previously mentioned study [41], this study proposes that the origin of cultivation correlated more closely to the anti-proliferative effects rather than the phenolic content or pigmentation level [35].

#### 3.2.2. Role of Pulse Bioactive Proteins

Protein isolates derived from pulses have also been investigated for their anti-proliferative effects. Mung bean protein extracts have been observed to have a stronger anti-proliferative effect than Adzuki bean extracts when using SKOVE and SMMC7721 cancer cells (IC_50_ of 505.1 and 323.6 µg/mL respectively) [43]. Other studies using protein fractions of large red (Nepalese) [44] and spotted beans [45] found that proliferation was inhibited in L1210 and MBL2 by both bean extracts. Spotted bean had a higher anti-proliferative effect compared to red bean with an IC_50_ value of 4 µM in L1210 and 9 µM in MBL2 cell lines. Moreover, protease inhibitor peptides from chickpea, kidney bean, mung bean, peas and lentils have also been investigated for their anti-proliferative potential [46]. From the five pulses examined, chickpea protease inhibitors were observed to be the most effective at inhibiting MDA-MB-231 proliferation, compared to other pulses.

Mung bean protein extracts have been demonstrated to inhibit proliferation in cancerous cells (MCF7 and Hela cells) while slightly inhibiting proliferation of normal fibroblast cells [47]. In addition, other in vitro investigations using various cancer cell lines have demonstrated anti-proliferative effects of isolated protein extracts including; Hemagglutinin—French bean [48], Homotetrameric—Haricot bean [49], Limynin—Lima beans [50], Coccinin—Scarlet bean [51], Mugoin—Mung bean [52], Lectin—Pinto beans [53], trypsin inhibitor—White cloud bean [54], Bowman-Birk inhibitors (BBI)—Peas [55], water-soluble protein extracts [56], and C25 protein fractions [57] from chickpea.

#### 3.2.3. Role of Short-Chain Fatty Acids

A limited number of studies have investigated the anti-proliferative effects of short chain fatty acids from pulses. The role of fatty acids in conjunction with coumarins from alhagi beans has been examined on C32 melanoma cells. The study found that short-chain fatty acids from alhagi beans inhibited proliferation with an IC_50_ value of 2700 mg/mL [58]. An in vivo analysis utilized Azoxymethane to induce colon cancer in rats [59]. The rats were fed with 75% Black bean and Navy bean short chain fatty acid, Butyrate. Rats fed with a diet of black bean or navy bean had reduced incidences of colon adenocarcinoma and total tumour multiplicity. The reduction in carcinogenesis from the diet was attributed to reduced body fat due to the presence of butyrate in the distal colon.

### 3.3. Pro-Apoptotic Effects of Pulses

#### 3.3.1. Pro-Apoptotic Bioactive Phenolic Compounds

In addition, to inhibition of inflammation and cancer cell proliferation, phenolic compounds from pulses have also been recognised as inducers of apoptosis (Table 2). The effect of faba bean phenolic extracts was examined on several human cancer cell lines [6]. The phenolic extracts were found to inhibit proliferation of cancer cells and specifically induce apoptosis in leukaemia cells while inhibiting angiotensin-converting-enzyme (ACE), α-glucosidase and pancreatic lipase. Studies have also investigated phenolic extracts of jamapa beans on HeLa and HaCaT cells [24,60]. Apoptotic induction in Hela cells was achieved by upregulation of pro-apoptotic proteins such as Bax and caspase3. Korean kidney bean phenolic extracts have also been identified to induce apoptosis through the Activated Protein Kinase (AMPK) signalling pathway [10]. This involved upregulation of proteins such as p-AMPK, p-Acc, p53 and p21 as a result of inhibition of AMPK by the interaction of compound C with AMPK activator 5-Aminoimidazole-4-carboxamide ribonucleotide (AICAR) [10].

Phenolic extracts from mung bean sprouts have been demonstrated to reduce inflammation, induce anti-proliferative effect and apoptosis in HeLa and HepG2 cells [61]. It was observed that the mung bean extracts upregulated TNFα and IFN-β, while inducing expression of IFNγ, apoptotic genes, tumour suppressor genes and inhibiting IL-4. A similar investigation has been conducted on phenolic extracts of lentil and pea sprouts [62]. The study revealed that in green non-sprouted and yellow sprouted pea, a 50% cytotoxicity was observed at 3 mg/mL [62]. Furthermore, in the sprouted green pea, sprouted lentil and non-sprouted lentils, induced cytotoxicity was seen at 6 mg/mL and in yellow non-sprouted pea at 7.5 mg/mL. The apoptotic effect of the extracts was linked to the induction of lactate dehydrogenase enzyme (LDH) release, DNA fragmentation and upregulation of caspase3 activity. An in vivo study incorporated red kidney bean phenolic extracts into female Sprague Dawley rats diet and found rats fed with the extract had lower mammary cancer incidences, multiplicity and tumour size [63]. The mechanism of action was identified as the mitochondrial pathway involving up-regulation of Bcl-2 associated X protein and down-regulation of B cell lymphoma 2 and X-linked inhibitor protein.

Furthermore, specific phenolic compounds responsible for activation of apoptosis have also been identified. For instance, flavonoid extracts from black bean hulls have been found to have pro-apoptotic effects on OCI-Ly7 lymphoma cells in mouse. It was believed that flavonoids have an effect on cell cycle by inducing cell cycle arrest at the S-phase and blocking progression to G2/M phase [64]. Isoflavones from chickpeas were observed to have cytotoxic effects at IC_50_ of 10–60 μg/mL as the extract upregulated cytoclasis, apoptotic body formation, caspase7, caspase9 and p53 [17]. It further showed a decrease in the following; P21, mitochondrial membrane potential, expression of Bcl-2-associated X protein but an increase in Bcl-2. Additionally, epigallocatechin and luteolin present in pea phenolic compound extracts have been linked to apoptotic activity induced in LS174, MDA-MB-453, A594 and K562 cancer cell lines [65]. The study found that these phenolic compounds were linked to darker pigmented pea varieties. Potential activation of apoptosis was identified via caspase3 pathway.

#### 3.3.2. Pro-Apoptotic Bioactive Peptides

Peptides extracted from pulses have been shown to exhibit a considerable effect in inducing apoptosis (Table 2). Non-digestible fractions from fermented beans of four different types including Azufrado higuera, Bayo madero, Negro 8025, and Pinto Durango have been examined for pro-apoptotic potential [66]. Extracts from the beans induced apoptosis in HCT116, RKO, and KM12L4 cancer cell lines. The investigation also established that extracts from all the four types exerted an anti-proliferative effect. Azufrado higuera upregulated cell cycle arrest markers such as p53 Ser^392^, p21 and downregulated cyclin-B1. Meanwhile, Bao Madero induced apoptosis through the mitochondrial pathway affected the transmembrane receptor TNFR1 and induced modification of markers including BAD, cytC, c-casp3, survivin and BIRC7 [66].

Further investigation isolated the aforementioned peptides from Large lima beans to examine the mechanistic pathways involved in inducing apoptosis by ACE inhibitor proteins [67]. The non-digestible fraction consisted of 70% protein, rich in ACE inhibitors, was sequenced as GLTSK, LSGNK, GEGSGA, MTEEY, and MPACGSS. It was demonstrated that the ACE inhibitor proteins GLTSK and GEGSGA were the most effective in inhibiting cell proliferation. GLTSK induced mitochondrial membrane disruption, by the loss of mitochondrial membrane potential and a 12.1-fold increase in reactive oxygen species. While GEGSGA initiated DNA damage by promoting poly-ADP-ribose polymerize (PARP) cleavage and halting cell cycle in G1phase. Consequently, activating oxaliplatin initiated the translocation of p53 protein in the apoptotic signal pathway. Similar mitochondrial dysfunction was observed when HCT116 and HT29 colorectal cancer cell lines were treated with hemagglutinin fraction from the black bean [68].

Furthermore, anti-proliferative activity of WKBL lectin from white kidney beans have been examined on HONE1, HepG2, MCF7 and WRL68 cancer cells [69]. The study showed that the anti-proliferative activity in HONE1 cells and HepG2 cells were high, while activity in MCF7 and WRL68 cells was considerably lower. Apoptosis was induced through the extrinsic pathway. The pathway involved upregulation of caspase 3, 8 and 9 in cells affected by WKBL treatment. Lectin found in lentils has demonstrated apoptotic activity through the extrinsic apoptotic pathway, the mechanism of action was through caspase 3, 8, and 9 as detected in CNE1 cells [70]. Additional apoptotic pathways were identified by phosphatidylserine externalization, mitochondrial depolarization and cell cycle arrest [70].

The anti-proliferative effect of proteins has also been investigated via the inhibition of glucose-regulated protein 78 (GRP78)—present only in cancerous cell membrane [71]. In the study, WIFPWIQL was formed using a GRP78 binding peptide and mung bean trypsin inhibitor protein. The synthesised protein was tested in vitro and in vivo. Cancer cell lines of HT29, SW620 and DLD1 exhibited apoptotic deaths while the normal cell line of FHC was unaffected. Xenografts in mice induced with human colorectal carcinoma also demonstrated reduced tumourigenesis. A potential pathway of apoptosis was through binding and activation of the constructed protein to GRP78, consequently, activating multiple apoptotic pathways and inducing G1 phase arrest [71].

#### 3.3.3. Pro-Apoptotic Pulse Short-Chain Fatty Acids

Anti-proliferative effects of short chain fatty acids have been associated with the induction of apoptosis [11,72,73]. Apoptotic induction by DNA fragmentation utilizing butyrate derived from black beans has been examined in HT29 cells [11] (Table 2). Further investigation indicated that the apoptotic effect was due to the modulation of RB1, CDC2, CDC25A, NFκβ ,and E2F genes [73]. In addition, an increase was observed in the pro-apoptotic genes APAF1, BID, CASP9, FASLG, TNFR10B, and Bcl2A. In vivo investigations of butyrate derived from black bean tested on male Sprague Dawley rats treated with 1.84 g/kg lyophilized samples induced substantial levels of apoptosis [72]. Lowering the number of total colonic Aberrant Crypt Foci initiated cell cycle arrest the G1 phase.

### 3.4. Anti-Metastatic Effects of Pulses

A limited number of studies have investigated the anti-metastatic potential of pulses. One such study examined the anti-metastatic potential of the chickpea, lentil, lupin, common bean, peas, faba bean, and cowpea, using HT29 cells [75]. It was observed that the albumin and globulin fractions from seeds inhibit cell proliferation at 100 µg/mL, with lupin and peas extracts displaying the highest and lowest anti-metastatic activity respectively. The study showed that, cell proliferation, correlated to anti-metastatic ability and that MMPIs present in these seeds to be novel metalloproteinase inhibitors. In addition, investigations on the anti-metastatic properties of pulses has been examined by evaluating the effect of protease inhibitor from field beans on mice injected with melanoma cells [76]. Two treatment approaches were investigated, firstly, melanoma cells were treated with extract pre-induction to mice and secondly by feeding mice with diets of 100 mg/kg of extract post induction of melanoma cells. Both approaches effectively reduced migration of metastatic lung melanoma cells by plasmin inhibitory action. Although the two studies have identified pulse protein factions to have potential anti-metastatic effect in vitro and in vivo, further research is warranted to understand the exact mechanism of action associated with MMP/protease inhibition.

## 4. Conclusions

**Figure 2:**
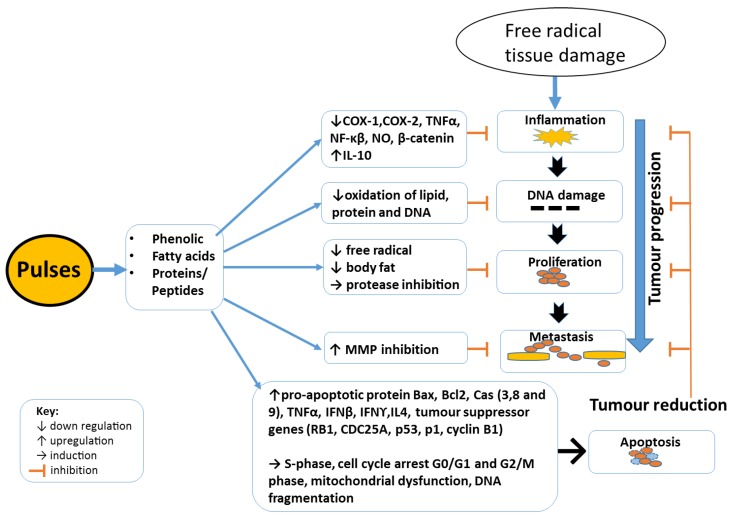
Potential interaction of pulse bioactive compounds with cancer pathways. Cyclooxygenase (COX), tumour necrosis factor α (TNFα), nuclear factor kappa β (NF-κβ), interleukin (IL), interferon (IFN), (LOX), nitric oxide (NO), matrix metalloproteinase inhibitors (MMPI).

The studies discussed in this review suggest that pulses may play an important role in reducing the risk of cancer occurrence and deterring its progression via diverse mechanisms. These mechanisms include reducing inflammation and cancer cell proliferation/metastasis as well as the induction of apoptosis in cancer cells. Figure 2 provides a summary of how bioactive compounds from pulses may play a role in the reduction of cancer development and progression. Although the studies outlined in this review demonstrate the potential anti-cancer properties of pulses, as well as the impact that processes such as sprouting and cooking, may have on these activities, more research is needed to confirm these activities using in vivo human clinical trials.

## Figures and Tables

**Table 1:** Anti-inflammatory potential of pulse bioactive compounds.

Sample	Compound	Cell Line/Model	Mechanisms/Outcomes of Anti-Inflammatory Effect	Reference
Black, spotted and common bean (*Phaseolus spp)*	Phenolics	HT-29 human colon cancer cells	Inhibition of pro-inflammatory proteins cyclooxygenase-2 (COX-2), TNFα and NF-κβ, and increased expression of anti-inflammatory protein IL-10.	[32]
Lentil (*Lens esculenta*) Faba bean (*Vicia faba)*	Phenolics		Faba bean hull extracts exhibited mild LOX inhibitory activity, while lentils inhibited 15-LOX, COX-1 and COX-2.	[14]
Pigmented and non-pigmented beans (dry) (*Phaseolus spp)*	Phenolics	Female Sprague Dawley rats	Exhibited lowed incidences of mammary cancer, cancer multiplicity and tumour burden.	[35]
White kidney bean *(Ph. vulgaris)* Round purple bean (*Phaseolus spp)*	Phenolics	RAW 264.7 macrophage cells	Anti-inflammatory effect via a reduction in LPS stimulated macrophages of cytokine mRNA expression and nitric oxide production.	[36]
Adzuki bean *(Ph. angularis)* Mung bean *(Vigna radiate)*	Phenolics- hull and cotyledon		Protease inhibition was highest in hull faction followed by wholegrain and cotyledon.	[31]
Mung bean *(Vigna radiate)*	Phenolics- vitexin and isovitexin	RAW 264.7 macrophage cells	Exhibited anti-inflammatory effects.	[8]
Black Bean (*Phaseolus spp)*	Protein hydrolysates	RAW 264.7 macrophage cell	Nitric oxide synthesis was inhibited when treated with protein hydrolysates from germinated beans	[33]
Chickpea *(Cicer arietinum)*		ICR male mice	Reduction in the oxidation of lipid, protein and DNA, downregulation of cyclooxygenase COX-2 and inducible nitric oxide synthase and oncogenic protein β-catenin.	[37]
Mung bean *(Vigna radiate)*	Gamma-aminobutyric acid (GABA)	Yac-1 and 4T1 lymphoma cells Female Balb/c mice	High anti-cancer cytokine levels, spleen T cell populations, splenocyte cytotoxicity, and spleen/tumour antioxidant levels. Mitotic divisions in spleen were also reduced along with antioxidant and nitric oxide levels.	[34]

Cyclooxygenase-2 (COX-2), tumour necrosis factor α (TNFα), nuclear factor kappa β (NF-κβ), interleukin 10 (IL-10), Lipoxygenase (LOX).

**Table 2:** Pro-apoptotic pathways initiated by pulse bioactive compounds.

Sample	Compound	Cell Line/Model	Mechanisms for Apoptosis	Reference
Black Bean (*Phaseolus spp*)	Phenolics—hulls extract and flavonoid fraction	OCI-Ly7 lymphoma cells mouse	Induced cell population to S-phase, increased the overall survival of mice fed with bean extracts by blocking progression to G2/M phase.	[64]
Black Bean (*Phaseolus spp*)	Phenolics	HeLa human cervical cancer cells HaCaT human premalignant keratinocytes	Reduced number of cells in the G0/G1 phase in comparison to control and induced apoptosis.	[60]
Black Bean (*Phaseolus spp*)	Phenolics	HeLa human cervical cancer cells	Methanol extracts induced apoptosis via upregulation of pro-apoptotic proteins, Bax and Caspase-3.	[24]
Mung bean sprouts (*Vigna radiate*)	Phenolics	HeLa human cervical cancer cells, HepG2 human liver cancer cells	Regulation of tumour necrosis factor (TNF-α), Interferon (FN-β, IFNγ), Interleukin (IL-4), apoptotic genes and tumour suppressor genes.	[61]
Red beans (dry) (*Phaseolus spp)*	Phenolics—isoflavones	Female Sprague Dawley rats	Increase in pro-apoptotic proteins BCL-2–associated X protein and reduction in inhibitory apoptotic protein B cell lymphoma 2 and X-linked inhibitor, hence induction of apoptosis was via the mitochondrial pathway.	[63]
Peas (*Cajanus cajan*)	Phenolics	Caco-2 human colon cancer cell	Apoptotic action was linked to the induction of lactate dehydrogenase (LDH) release, DNA fragmentation and upregulation of caspase-3 activity.	[62]
Lentil (*Lens esculenta*)				
Peas (*Cajanus cajan*)	Phenolics—epigallocatechin and luteolin	LS174 human colon adenocarcinoma, MDA-MB-453 human breast carcinoma, A594 human lung carcinoma, K562 myelogenous leukaemia	Induction of caspase 3 pathway.	[65]
Chickpea (*Cicer arietinum*)	Phenolics—isoflavones	SKBr3, MCF-7 human breast cancer cells	Upregulation of cytoclasis, apoptotic body formation, caspase 7, caspase 9, P53, and P21 decrease in mitochondrial membrane potential, expression of Bcl-2-associated X protein and increased Bcl-2.	[17]
Korean kidney bean husk (*Ph. vulgaris*)	Phenolics	HT-29 human colon cancer cells	Upregulation of proteins adenosine monophosphate-activated protein kinase (p-AMPK), protein acetyl-CoA carboxylase (p-ACC), p53 and p21.	[10]
Speckled lentil (Lens esculenta; *Ervum lens*)	Peptide-lectin	Nasopharyngeal carcinoma CNE1 and CNE2 cells	Phosphatidylserine externalization, mitochondrial depolarization and cell cycle arrest. An extrinsic apoptotic pathway involving caspase 3, 8, and 9 were also detected in CNE1 cells	[70]
Black Bean (*Phaseolus spp*)	Peptide—hemagglutinin	HCT116, HT-29 human colon cancer cells	Hemagglutinin successfully penetrate the cytoplasm of colorectal cancer cells and instigate mitochondrial dysfunction and apoptotic activity	[68]
Mung bean (*Vigna radiate*)	Peptide—GRP78 binding peptide WIFPWIQL and the active fragment of mung bean trypsin inhibitor	HT-29, SW620, DLD1 human colon cancer cell, FHC human normal colon cells, female mice with severe combined immune deficiency (SCID/NOD)	Activation of multiple apoptotic pathways and induction of G1 phase arrest.	[71]
White kidney bean (*Ph. vulgaris*)	Peptide—WKBL (lectin)	HONE1 epithelial tumour cells	Caspase 3, 8 and 9 were upregulated in cells as a result of WKBL treatment outlining apoptosis as the mechanism of action following an extrinsic pathway.	[69]
		HepG2 human liver cancer cells		
		MCF-7 human breast cancer cells		
		WRL68 human normal adherent cells		
Black, spotted and common bean (*Phaseolus spp*)	Peptides	HCT116, RKO, KM12L4 human colorectal cancer cells	P53 expression was up-regulated along with modification of p21 and cyclin B1	[66]
Large Lima beans (*Ph. lunatus*)	Peptides—GLTSK, LSGNK, GEGSGA, MTEEY, and MPACGSS	HCT116, CCD-33Co human normal colon cells	GLTSK caused mitochondrial membrane disruption via loss of mitochondrial potential (Δψm) and increased intracellular ROS. GEGSGA caused DNA damage via cleavage of PARP and cell cycle arrest in G1 phase, suggesting oxaliplatin initiated activation and nuclear translocation of p53.	[67]
Black Bean (*Phaseolus spp*)	Short-chain fatty acid	HT-29 human colon cancer cells	Modulation of: RB1, CDC2, CDC25A, NFKB and E2F genes; and pro-apoptotic genes: APAF1, BID, CASP9, FASLG, TNFR10B and BCL2A genes.	[73]
Black Bean (*Phaseolus spp*)	Short-chain fatty acids—butyrate	Male Sprague Dawley rats	Rats demonstrated apoptotic effects and cell cycle arrest in G1 phase.	[72]
Black Bean (*Phaseolus spp*)	Short-chain fatty acids—butyrate	HT-29 human colon cancer cells	DNA fragmentation induced by the extracts indicating that cells were undergoing apoptosis.	[11]
Bean (*Phaseolus spp*)	Polysaccharides—human gut fermented bean	HT-29 human colon cancer cells	Extract induced apoptosis via modulation of 72 p53-mediated signal transduction response genes in human colorectal cancer cells	[74]
